# New insights into the effects of ethylene on ABA catabolism, sweetening and dormancy in stored potato tubers

**DOI:** 10.1016/j.postharvbio.2020.111420

**Published:** 2021-03

**Authors:** R. Tosetti, A. Waters, G.A. Chope, K. Cools, M.C. Alamar, S. McWilliam, A.J. Thompson, L.A. Terry

**Affiliations:** a Plant Science Laboratory, Cranfield University, Cranfield, MK43 0AL, UK; b PepsiCo Inc., 1991 Upper Buford Circle, St. Paul, MN 55108, USA; c PepsiCo International Limited, Beaumont Park, 4 Leycroft Road, Leicester, LE4 1ET, UK; d Postharvest BioScience Consultant, Binfield, Berkshire, RG42 5LG, UK

**Keywords:** Exogenous ethylene, *Kunitz-type invertase inhibitor*, ACO, ACS, 1-MCP

## Abstract

Continuous ethylene supplementation suppresses postharvest sprouting, but it can increase reducing sugars, limiting its use as an alternative to chlorpropham for processing potatoes. To elucidate the mechanisms involved, tubers were treated after curing with or without the ethylene binding inhibitor 1-methylcyclopropene (1-MCP at 1 μL L^−1^ for 24 h), and then stored in air or air supplemented with continuous ethylene (10 μL L^−1^). Across three consecutive seasons, changes in tuber physiology were assessed alongside transcriptomic and metabolomic analysis.

Exogenous ethylene alone consistently induced a respiratory rise and the accumulation of undesirable reducing sugars. The transient respiratory peak was preceded by the strong upregulation of two genes encoding 1-aminocyclopropane-1-carboxylate oxidase (ACO), typical of wound and stress induced ethylene production. Profiles of parenchymatic tissue highlighted that ethylene triggered abscisic acid (ABA) catabolism, evidenced by a steep fall in ABA levels and a transient rise in the catabolite phaseic acid, accompanied by upregulation of transcripts encoding an ABA 8ˊ-hydroxylase. Moreover, analysis of non-structural carbohydrate-related genes revealed that ethylene strongly downregulated the expression of the *Kunitz-type invertase inhibitor*, already known to be involved in cold-induced sweetening. All these ethylene-induced effects were negated by 1-MCP with one notable exception: 1-MCP enhanced the sprout suppressing effect of ethylene whilst preventing ethylene-induced sweetening.

This study supports the conclusions that: i) tubers adapt to ethylene by regulating conserved pathways (*e.g.* ABA catabolism); ii) ethylene-induced sweetening acts independently from sprout suppression, and is similar to cold-induced sugar accumulation.

## Highlights

• Continuous ethylene activates ACO transcription and respiration in potatoes.
• 1-MCP completely inhibits the respiratory rise induced by continuous ethylene exposure.
• Exogenous ethylene promptly activates parenchymatic ABA catabolism *via* CYP707A upregulation.
• A *Kunitz-type invertase inhibitor* mediates ethylene-induced sweetening.
• A physiologically targeted ethylene + 1-MCP treatment offers an alternative to CIPC.

## 1 Introduction

Maintaining dormancy and low reducing sugars content during storage are key priorities for the global potato processing industry. Control of dormancy during commercial storage is overly reliant on a longstanding synthetic sprout suppressant, chlorpropham (CIPC), with 85 % of ware potatoes treated in the UK alone (Hinchcliffe et al., 2018). Over the last 10 years, CIPC has been under regulatory scrutiny, and in June 2019 the European Union legislated for its withdrawal, such that the 2019−2020 season was the last storage season where the application of CIPC will be permissible. Among the alternative sprout suppressant strategies available, continuous ethylene supplementation has received much attention (Daniels-Lake et al., 2005, 2011; Foukaraki et al., 2014, Foukaraki et al., 2016a, 2016b; Prange et al., 1998, 2005; Rylski et al., 1974; Suttle, 1998), and in 2003 the Chemical Residue Directorate (UK) approved its use as a sprout suppressant (Alamar et al., 2017a). The inhibition of potato sprout growth by ethylene is well-known; and similar effects of using ethylene to extend ecodormancy have been described in other low-ethylene-producing storage organs, like onion bulbs (Cools et al., 2011; Ohanenye et al., 2019) and sweetpotato roots (Amoah et al., 2016). Morover, continuous ethylene supplementation induced an accumulation of abscisic acid (ABA) in potato tubers (Foukaraki et al., 2016a), and has been associated with tuber dormancy (Alamar et al., 2017a; Sonnewald and Sonnewald, 2014).

Despite the positive effect on extending dormancy, ethylene is also known to elicit the accumulation of reducing sugars (Daniels-Lake et al., 2005; Foukaraki et al., 2016a, 2016b), similarly to storage temperature below 7 °C (Bhaskar et al., 2010; Liu et al., 2013). Thus, ethylene-induced and low temperature induced sweetening can result in darker fry colour and associated acrylamide formation during high temperature (>120 °C) cooking. Whilst low temperature sweetening can be prevented by storing tubers at temperatures above 7 °C, ethylene-induced accumulation of reducing sugars can be suppressed with varying degrees of success: using 1-MCP prior to ethylene treatment (Daniels-Lake et al., 2008; Foukaraki et al., 2016b; Prange et al., 2005); by a gradual introduction of ethylene into the atmosphere known as ramping (Colgan et al., 2014); or by physiologically targeting ethylene application (Foukaraki et al., 2014). Nevertheless, the success of these strategies relies strongly on many factors (such as treatment dose, tuber age, and cultivar). Indeed, while the dual effect of ethylene on sprouting and sugar accumulation have been extensively described, the underlying mechanisms involved are poorly understood.

Potato tubers produce very low levels of ethylene during storage, and it is not clearly established whether endogenous ethylene is part of an ecologically important mechanism for tuber dormancy, or whether tubers have simply retained exploitable machinery for an ethylene response common to most plant tissues. The aim in this study was to understand how, and through which regulatory molecular pathways, exogenous ethylene and/or 1-MCP elicited their effects on potato tuber quality. Physiological, biochemical, and transcriptional changes of pathways of interest (ethylene, ABA, and sugar metabolism) induced by exogenous ethylene were therefore investigated.

## 2 Materials and methods

### 2.1 Plant material and storage conditions

Across three seasons (Year 1: 2016−17; Year 2: 2017−18; Year 3: 2018), UK-grown tubers of cvs. VR808 (long storage) and Shelford (short storage) cultivars were sourced from two diverse geographical locations (Yorkshire and Cambridge) and transferred to Sutton Bridge Crop Storage Research (Agriculture and Horticulture Development Board, Sutton Bridge, Lincolnshire, UK) after harvest for curing (Table 1). Immediately after arrival, tubers were passed over a grading line to remove loose soil and rotten, damaged, green and/or undersized (<45 mm) tubers. After curing, tubers were either treated at curing temperature (Table 1) with 1-MCP (M-), 1 μL L^−1^for 24 h (Foukaraki et al., 2016b), or kept in air (24 h. Control, C-) in storage chambers at Sutton Bridge, following standard operating procedures. CIPC was not applied to tubers used in this study.

**Table 1:** Experimental design and storage conditions. Treatments were applied as individual treatments or in combination (CC, CE, MC, ME).

Year and growing location	Cvs.	Harvest		Curing T°C and duration	Treatments		Storage T°C	Storage start	Ethylene start	Sprout assessment	Sampling	Storage end and duration
Year b, 2016/17 Yorkshire	VR808, Shelford^a^		10 Oct	14 °C 6 d	± 1-MCP^b^ 19 Oct	1 μL L^−1^, c4 h	8.5 °C	07 Nov	15 Nov	Weekly: Nov-Dec; Monthly: Jan-Feb	Every 2−3 days from ethylene start in Nov-Dec	27/03/17 VR808 141 days
					± Ethylene^c^	10 μL L^−1^, continuous					Bulk and tissues	27/02/17 Shelford 113 days
Year c, 2017/18 Yorkshire	VR808, Shelford^a^		03 Oct	13 °C 8 d	± 1-MCP^b^ 17 Oct	1 μL L^−1^, c4 h	8.5 °C	03 Nov	06 Nov	Weekly: Nov-Dec; Monthly: Jan-Feb	Daily during the first week in ethylene; weekly in Nov-Dec. Bulk and tissues	05/02/18 VR808 91 days
					± Ethylene^c^	10 μL L^−1^, continuous						27/11/17 Shelford 24 days
Year 3, 2018 Cambridge	VR808		10 Oct	12.5 °C 8 d	± 1-MCP^b^ 18 Oct	1 μL L^−1^, c4 h	8.5 °C	02 Nov	05 Nov	Weekly	Weekly	14/12/18 40 days
					± Ethylene^c^	10 μL L^−1^, continuous					Bulk and tissues	

a Shelford tubers have been used as trigger for the start of ethylene treatments, data are not discussed in the paper.

b Applied at Sutton Bridge at curing temperature.

c Applied at Cranfield University at storage temperature.

After 1-MCP treatment, tubers were ventilated for 24 h and then transferred to Cranfield University (Bedfordshire, UK) within 2 h whereupon they were promptly sorted across treatments and stored in gas-tight boxes (100 L, water-sealed) inside variable temperature rooms. The boxes were continuously flushed (5 L min^−1^) with air (Control, -C) or ethylene (-E), 10 μL L^−1^, as previously described (Amoah et al., 2017; Collings et al., 2018).

**Fig. 1:**
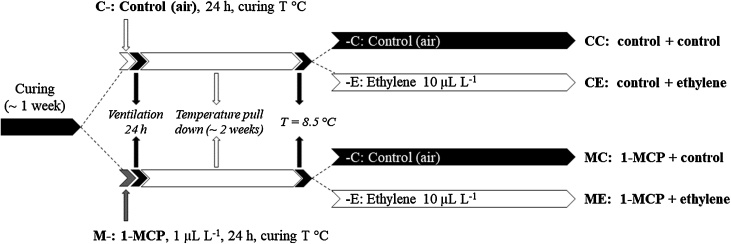
Ethylene treatments. Potato tubers cv. VR808 were stored at 8.5 °C under four two-stage treatments for pre-storage and storage: CC (control + control); CE (control + continuous ethylene); MC (1-MCP + control); ME (1-MCP + continuous ethylene). 1-MCP (1 μL L^-1^) was applied at the curing temperature (12.5 - 14 °C. Table 1).

The experiments followed a completely randomized design with three replicates (boxes) per treatment/cultivar. To minimize chilling stress and allow time for wound healing, tubers underwent a controlled cooling regime from the curing temperature (Table 1), at a rate of 0.3 °C reduction per day at ambient relative humidity (RH), to a holding temperature of 8.5 °C, when the first sprout assessment was carried out. The treatment combinations for each year were: CC, control + control; CE: control + ethylene; MC: 1-MCP + control; ME: 1-MCP + ethylene (Fig. 1).

### 2.2 Sprout assessment and ethylene treatment

**Fig. 2:**
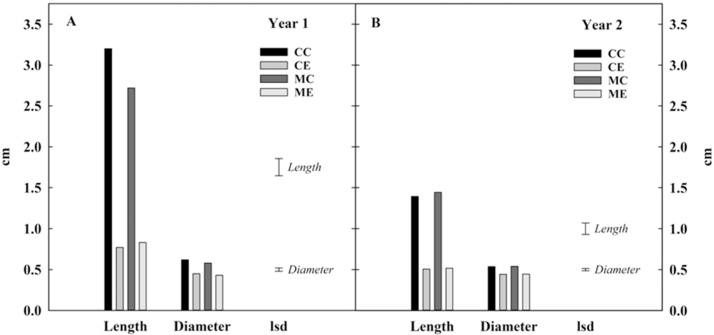
Sprout vigour (length and diameter) at the end of storage. Potato tubers cv. VR808 were stored at 8.5 °C under four different treatments: CC (control + control); CE (control + continuous ethylene, 10 μL L^−1^); MC (1-MCP, 1 μL L^−1^ 24 h, + control); ME (1-MCP + continuous ethylene, 10 μL L^-1^ ethylene). Year 1, storage duration 141 days. Year 2, storage duration 91 days. Least significant difference (LSD_0.05_) bars are shown.

Sprout assessment was carried out on 40 tubers per replicate/treatment, and sampling point. Tubers were ranked according to the following categories: no eye movement; a: peeping; b: sprout < 1 mm; c: 1 mm < sprout < 3 mm; d: sprout > 3 mm. For each season, the first sprout assessment was done as soon as the temperature reached the final storage temperature of 8.5 °C; thereafter, tubers were assessed at regular intervals (Table 1). At the end of the storage (Table 1), sprout vigour (length and diameter) was recorded (Fig. 2).

Continuous ethylene supplementation (10 μL L^−1^) was started at the same time for all the cultivars; when any cultivar showed 10 % of tubers peeping then ethylene treatment commenced (Foukaraki et al., 2016b). In each year, cv. Shelford tubers showed 10 % peeping from the beginning of storage (day 0), and as a consequence ethylene was initiated as soon as the temperature reached 8.5 °C. Ethylene concentration inside the boxes was checked using a gas chromatograph (Agilent) fitted with a packed Chromosorb column (Chromosorb 103, 112–149 (60/80 mesh), 2 m x 2 mm, stainless steel) and flame ionisation detector (FID). Ethylene concentration was quantified by comparing areas under peaks in the chromatograms to calibrations performed with certified gas (10.1 μL L^−1^; BOC, Surrey, UK).

### 2.3 Continuous *in situ* respiration rate measurements

Immediately after the arrival at Cranfield University and prior to the temperature pull down, small batches of tubers, *circa* 6 kg per treatment, were washed with tap water and air-dried before being stored inside 16 L boxes (Year 1, two cultivars.; 3 boxes with air and 3 boxes with ethylene each cultivar – Sh, cv. Shelford; VR, cv. VR808; -C:CC + MC, -E:CE + ME. Years 2 and 3, one cultivar, 3 boxes/treatment – VR, VR808; CC, CE, MC, ME. Fig. 1) at the same condition of 100 L boxes (flow16 L _box_ = 0.5 L min^−1^). The 16 L boxes were connected to a highly sensitive respirometry system (Sable Systems, NY, USA), and respiration rates were monitored from the beginning of storage (8.5 °C). Respiration rates were measured continuously for two weeks *in situ* (storage room at 8.5 °C), and for all treatment combinations, according to Alamar et al. (2017b).

### 2.4 Sampling

**Fig. 4:**
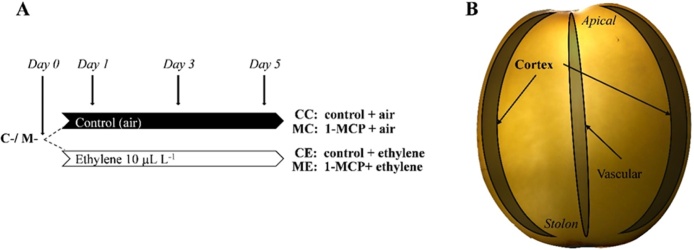
Experimental design of cortex sampling for RNAseq and LC/MS–MS analysis. Potato tubers cv. VR808 were stored at 8.5 °C under four different treatments: CC (control + control); CE (control + continuous ethylene, 10 μL L^-1^); MC (1-MCP, 1 μL L^-1^ 24 h, + control); ME (1-MCP, 1 μL L^-1^ 24 h + continuous ethylene, 10 μL L^-1^). A, sampling time. B, Potato slice showing individual tissues (cortex and vascular) sampled.

After sprout assessment, bulk (representing the whole tuber) and individual tissue (cortex and vascular) samples were collected for every treatment combination, at regular intervals (Table 1). Bulk samples consisted of two opposite wedges (including skin) from three different tubers belonging to the same box (1 rep = 6 wedges), were pooled together as one replicate. Cortex and vascular samples were also collected from the same tubers used for the bulk samples (Fig. 4). A central longitudinal slice of each tuber, 5−6 mm width was peeled, and vascular, and cortex (excluding vascular ring) tissues were isolated with a scalpel blade. All samples were immediately snap-frozen in liquid nitrogen and freeze-dried in readiness for the further analysis.

### 2.5 Sugar analysis

Sucrose, glucose, and fructose from the freeze-dried samples (bulk and individual tissues) were extracted as described by Foukaraki and colleagues (2016a, 2016b). The extracts were analysed using an Ion Chromatography system by PepsiCo (IC3000 with CarboPacPA20 column, and IC5000 with CarboPacSA10 microbore column. Dionex). The extracts were diluted (1:10, 1:50, 1:100 depending on the sample) prior to the injectionwith xylose used as an internal standard. Sugar concentrations were calculated against authentic calibration standards of fructose, glucose, and fructose and expressed as g kg^−1^ DW.

### 2.6 Transcriptomics

Cortex samples from year 2 were selected to investigate the transcriptomic variations induced by each treatment. Total RNAs were isolated from samples collected during the first week in ethylene (Fig. 4). The RNA extraction was performed on *circa* 15 mg (±1 mg) of freeze-dried material using CTAB buffer (Untergasser, 2008), before concluding using the RNeasy plant Kit (Qiagen) following the manufacturers procedures for RNA binding, on-column DNAse treatment (Qiagen), and elution. Before sending the samples for sequencing, quantity and integrity of nucleic acids were checked with the Genova Nanodrop (Fisher) and agarose gel (1.5 %) electrophoresis.

The samples were sent to Earlham Institute (Norwich, UK) for Illumina RNAseq library preparation and sequencing in two batches (TruSeq® Stranded Total RNA Library Prep Plant and NEBNext® Ultra™ II Directional RNA Library Prep Kit, respectively. Illumina). Samples were sequenced using Illumina HiSeq4000 platform which produced paired end 150 bp reads (150 PE), and NovaSeq 6000 SP ﬂow cell which also produced 150PE reads, respectively. The quality of the generated reads was assessed using FastQC (Andrews, 2010) before and after the trimming of Illumina adapters and removal of low-quality reads with LEADING:20 TRAILING:20 SLIDINGWINDOW:4:20 MINLEN:50 as options in Trimmomatic (Bolger et al., 2014).

Reads were aligned to version 4.04 of the double monoploid (DM) potato reference (Hardigan et al., 2016) using TopHat2 aligner (Kim et al., 2013) using -g 20 to allow for multi-mapping. Uniquely mapping reads were kept by filtering out all multi-mapped reads using BamToolsfilter (Barnett et al., 2011). Read counts per genes were calculated using HTSeq count union option (Anders et al., 2015). Normalized expression values (fragments per million per kilobase -fpkm) were calculated using a custom *perl* script for all samples, then genes were annotated using the version 3.4 of the DM potato genome reference. Differential expression analysis was performed with DESeq2 (Love et al., 2014) applying cut-offs of at least a 2-fold change in normalised expression and adjusted p-value < 0.05.

The individual normalised expression values were transformed into z-scores, subtracting the gene expression mean, and dividing by the gene expression standard deviations. The transformed datasets were evaluated and visualised in heat maps using Morpheus software (https://software.broadinstitute.org/morpheus). Pearson’s correlation analysis and hierarchical clustering (Morpheus default parameters for cluster metric and linkage method) were applied to both the complete dataset, and to specific genes relating with pathways of interest (ethylene, abscisic acid, starch-sugar metabolism, Supplemental Table 1). Genes for the specific pathways were selected using the provided annotation in the version 3.4 of the DM potato reference, and the respective z-score visualised in heat maps. Then, genes exhibiting differential expression were individually investigated through ANOVA.

### 2.7 Plant growth regulators

The samples selected for the transcriptomics and a second batch of cortex samples from year 1 (storage day 0, 2, and 4) were used for ABA and ABA metabolites analysis. Freeze-dried powdered potato cortex (5.0 ± 0.1 mg) was extracted with 500 μL of an ice-cold HPLC grade methanol:water:formic acid (60:35:5 v/v/v) solution, following Müller and Munné-Bosch (2011) with modifications. The labelled forms of the compounds [2H3]-dihydrophaseic acid (-)-7′,7′,7′-d3 dihydrophaseic acid (d3-DPA);; [2H3]-phaseic acid (-)-7′,7′,7′-(d3-PA); and [2H4]-abscisic acid (-)-5,8′,8′, 8′-(d4-ABA) were added to the mixture as internal standards at a concentration of 1 μg L^−1^. A blank (internal standard and extraction solvent) and a double blank (only extraction solvent) were considered every 12 samples. The samples were first placed in a star-beater (VWR, Leics., UK) at 30 Hz for 2 min, and then they were kept on ice and in the dark for 20 min before centrifuging at 24 x g for 10 min at 4 °C. Following centrifugation, the extracts were freeze-dried overnight to remove the extraction solvent. Samples were reconstituted with 500 μL of ice-cold HPLC grade methanol:water:formic acid (60:35:5 v/v/v), vortexed and sonicated for 20 s. After centrifugation (24 x g for 10 min at 4 °C) the supernatant was collected and filtered through a 0.2 μm nylon filter and collected in silanised amber HPLC vials.

Endogenous plant growth regulator concentrations were quantified according to Morris et al. (2019) with slight modifications, using a LC/MS-MS instrument with an Agilent 1200 series HPLC system (Agilent, Berks., UK) coupled to a Q-Trap 6500 mass spectrometer (AB Sciex, MA, USA). The extracts were injected onto a Phenomenex Luna 3 μm C18 100 × 2 mm column with guard column at 40 °C. The mobile phase was based on two solutions: (A) 2 % acetonitrile in 10 mM ammonium formate, and (B) 95 % acetonitrile in water with 0.1 % formic acid, using an increasing gradient of B (2 % for 4 min, 16 % at 20 min and 34.5 % at 25 min) at a flow rate of 200 μL min^−1^. The presence and abundance of hormones was calculated by comparing sample peak areas to each standard calibration curve as relative response between internal standards and standard.

### 2.8 Statistical analysis

The data from respiration, sugar, ABA and ABA metabolites, and selected genes normalised expression were analysed with Statistica software (v.12 and v.13) with the appropriate ANOVA approach for the experimental design (*e.g.* repeated measures ANOVA for respiration). The Least Significant Difference was calculated with a p < 0.05. When the interaction between treatment and time was not significant the Standard Error was represented (p < 0.05). RNAseq data were analysed with R package Limma (Bioconductor), and Morpheus was used for Pearson’s correlation and hierarchical clustering analysis and visualisation (https://software.broadinstitute.org/morpheus).

## 3 Results

### 3.1 Treatment effect on tuber respiration, sprout vigour, and reducing sugars content

**Fig. 3:**
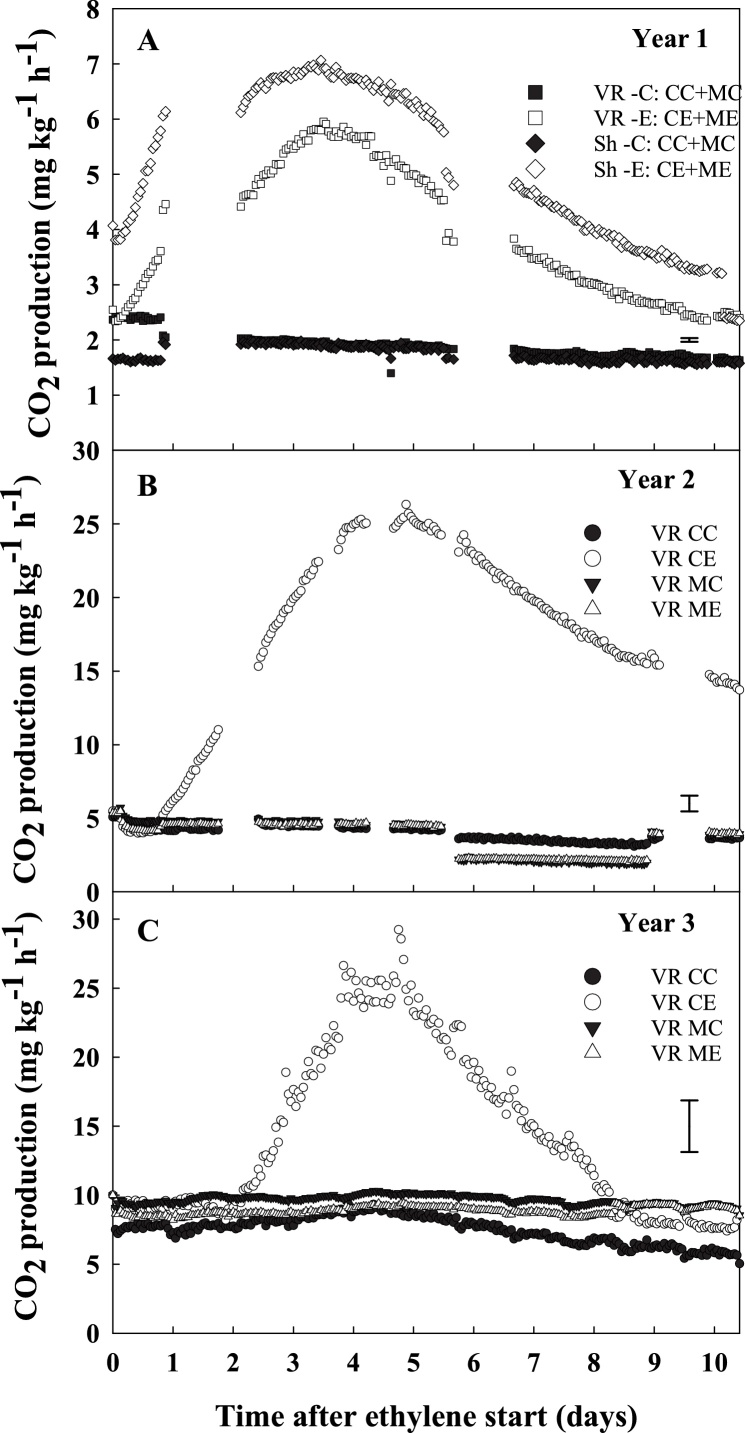
Real time respiration activity (CO_2_ production, mg kg^−1^ h^−1^). Potato tubers cvs. VR808 (VR) and Shelford (Sh year 1) were stored at 8.5 °C under four different treatments: CC (control + control); CE (control + continuous ethylene, 10 μL L^-1^); MC (1-MCP, 1 μL L^-1^ 24 h, + control); ME (1-MCP + continuous ethylene, 10 μL L^-1^). In year 1, tubers pre-treated with and without 1-MCP (1 μL L^-1^24 h)were mixed in equal quantities so give combined treatments CC + MC and CE + ME. The vertical bars represent the Standard Error as calculated by repeated measures ANOVA.

Respiration was monitored *in situ* throughout storage: within seventy-two hours from the start of continuous ethylene supplementation (10 μL L^−1^), ethylene-treated tubers showed a transient increase in CO_2_ production, which peaked at five days before declining, whereas no increase was detected in the absence of ethylene (Fig. 3).

Preliminary analysis of respiration rate in year 1 focused on the sole effect of storage treatment (-E *vs*. -C), regardless of the pre-storage treatment (C- or M-): an equal number of C- and M- tubers within the same cultivar (cvs. Shelford and VR808) were pooled together and supplied with air or air and ethylene (-C and -E, respectively). Both cultivars showed a 3-fold increase in respiratory activity after ethylene supplementation (Fig. 3A). In years 2 and 3, to elucidate the additional effect of 1-MCP pre-storage treatment, the analysis focused on each individual treatment, but for cv. VR808 only (Fig. 3B and C). In the absence of the 1-MCP pre-storage treatment, ethylene had a strong and transient effect on respiration rate (Fig. 3B and C, CE treatment) whilst 1-MCP pre-storage treatment completely negated this response (ME treatment). The respiration peak due to ethylene treatment of cv. VR808 was approximately 4.5-fold lower in year 1 compared to years 2 and 3; a lower response would be expected in year 1 where half the tubers had received the 1-MCP pre-storage treatment.

**Fig. 5:**
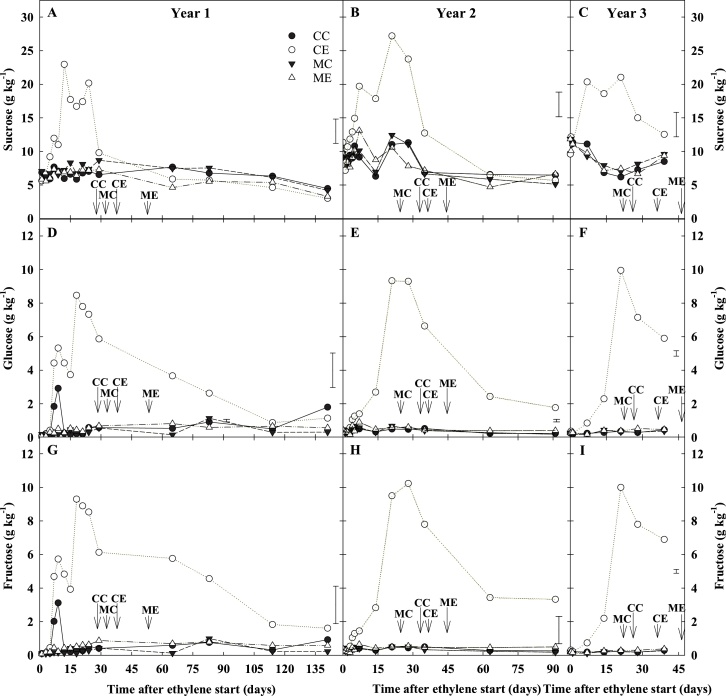
Sugar content (DW g kg^−1^) of bulk samples. Potato tubers cv. VR808 were stored at 8.5 °C under four different treatments: CC (control + control); CE (control + continuous ethylene, 10 μL L^-1^); MC (1-MCP, 1 μL L^-1^ 24 h, + control); ME (1-MCP + continuous ethylene, 10 μL L^-1^). Sucrose (A, B, C). Glucose (D, E, F). Fructose (G, H, I). Arrows indicate the time of dormancy break (10 % peeping) in each treatment. Least significant difference (LSD_0.05_) bars are shown.

Differences in respiration activity were transient and lasted one-to-two weeks from the start of continuous ethylene treatment. In contrast, long-term ethylene supplementation reduced sprout vigour, irrespective of 1-MCP pre-treatment (Fig. 2), and increased sugar accumulation (Fig. 5). Whilst, 1-MCP pre-treatment did not further reduce sprout elongation caused by continuous ethylene in storage (ME *vs*. CE) (Fig. 2), it did induce a one-to-three weeks extension of ecodormancy compared to CE in all three years (Fig. 5, arrows. Supplemental Fig. 1).

Ethylene elicited increased sucrose, glucose, and fructose concentrations from the first weeks of storage in CE tubers (Fig. 5) relative to all other treatments. Sucrose content in bulk samples exhibited a transient increase in CE tubers during the first one-to-three weeks of storage, while no variations were observed among all other treatments (Fig. 5A-C). Sucrose concentrations in CE tubers dropped within five weeks of storage, and no significant differences among treatments were identified thereafter. Glucose and fructose peaked in CE tubers around the third week of storage in ethylene, and were *ca.* 10 times higher than at the beginning of storage (Fig. 5D-I). Following the ethylene-induced accumulation of sugars in CE tubers, a decrease in glucose and fructose contents was also observed; however, this decline was more gradual and slightly delayed compared to sucrose (Fig. 5). CE tubers showed at least four-fold greater reducing sugar concentrations compared to other treatments for the first three months of the storage period; thereafter the differences among treatments were lost (Fig. 5D–E, G–H). The application of 1-MCP completely inhibited ethylene-induced sugar accumulation, and indeed no differences in sugar profiles were found among ME, MC, and CC. Taken together, these data suggested that the long-lasting increase in reducing sugars and delay in sprout growth may depend on the initial responses triggered by exogenous ethylene. The impact of ethylene supplementation on the individual sugar profile (rapid accumulation, short plateau and slow decline) seemed to indicate a regulated and sequential activation of sugar metabolism under ethylene supplementation.

### 3.2 Transcriptional changes induced by exogenous ethylene in potato cortex tissue

**Fig. 6:**
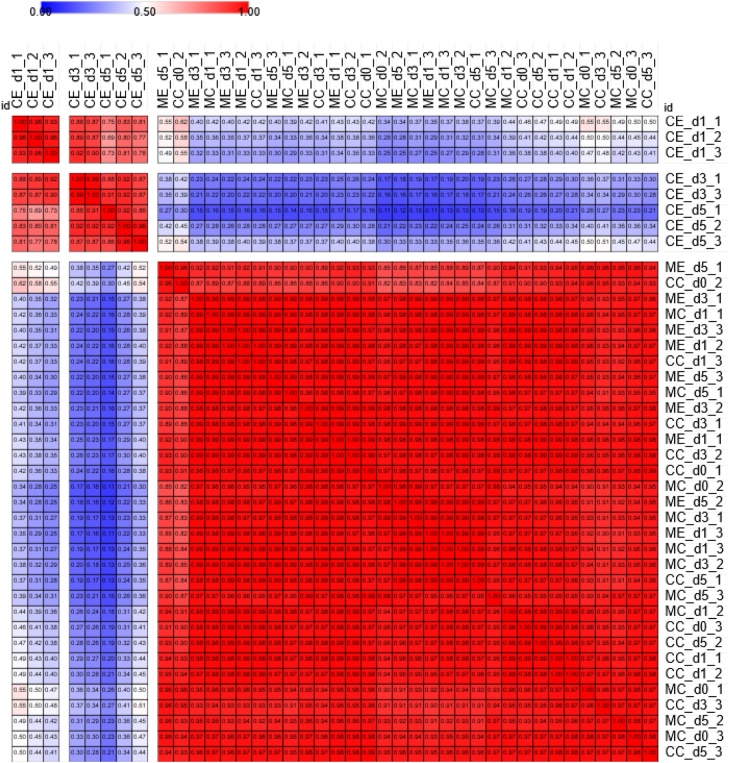
Pearson’s correlation of global transcriptomic data. Cortex was sampled from cv. VR808 tubers stored at 8.5 °C under four different treatments: CC (control + control); CE (control + continuous ethylene, 10 μL L^−1^); MC (1-MCP, 1 μL L^−1^ 24 h, + control); ME (1-MCP + continuous ethylene, 10 μL L^−1^). d0, day 0. d1, day 1. d3, day 3. d5, day 5. The number following the day indicates the replicate. The similarity matrix is separated according to the three hierarchical clusters identified (Supplemental Fig. 2).

Cortex samples collected during the first five days of ethylene exposure in year 2 (Fig. 4) were selected to investigate the transcriptional differences between CC, CE, MC and ME treatments. Separation between CE and the other treatments was clearly observed, whilst it was not possible to differentiate among CC, MC, or ME (Fig. 6). ME transcriptomic changes clustered, and correlated strongly with those of the MC samples, but were divergent from CE samples, similarly to what was observed for respiratory activity and reducing sugar accumulation analysis. Within CE samples, it was possible to identify two sub-clusters, one limited to day 1, and the other encompassing both days 3 and 5.

Due to the complexity of the experimental design, and the high number of possible contrasts resulting from the differential expression analysis (between and within treatments), further investigations were carried out on specific pathways of interest including ethylene, ABA, and starch/sugar metabolism.

### 3.3 Ethylene responses in cortex tissue: ethylene pathways

**Fig. 7:**
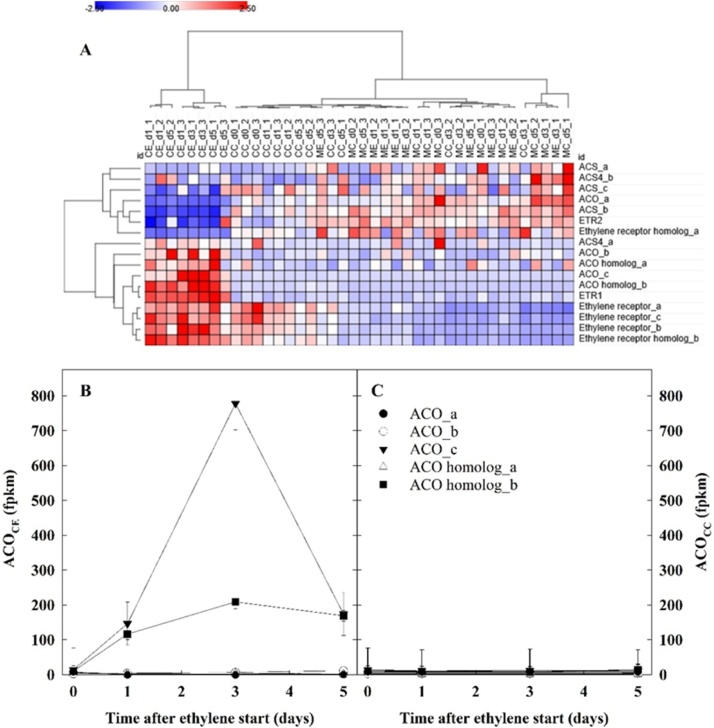
Ethylene biosynthesis and signalling. Cortex was sampled from cv. VR808 tubers stored at 8.5 °C under four different treatments: CC (control + control); CE (control + continuous ethylene, 10 μL L^−1^); MC (1-MCP, 1 μL L^−1^ 24 h, + control); ME (1-MCP + continuous ethylene, 10 μL L^−1^). Heat map of selected ethylene biosynthesis and signalling genes (A); and gene expression *vs*. time plots for *ACO* genes for CE tubers (B) and CC tubers (C). Gene expression is reported from RNAseq data, where fpkm is fragments per kilobase of transcript per million mapped reads. Error bars representing the Standard Error (B, C) are shown. ACS*, 1-aminocyclopropane-1-carboxylate synthase*; ACO*, 1-aminocyclopropane-1-carboxylate oxidase;* ETR1*, Ethylene Receptor 1*.

The effect of continuous ethylene supplementation on the ethylene biosynthesis and signalling pathway was evaluated by analysing the normalised expression levels of genes selected based on annotation (Fig. 7A). CE tubers exhibited an overall downregulation of *ACS* (*1-aminocyclopropane-1-carboxylate synthase*) genes, while *ACO* (*1-aminocyclopropane-1-carboxylate oxidase*) family showed an upregulation of expression in response to ethylene. However, exceptions were found: *ACO_a*, was inhibited in CE samples and *ACS_4* did not show differential expression among treatments. Ethylene-induced expression was reported also for *ETR1* (*Ethylene Receptor 1*, PGSC0003DMG400007843) and another four genes annotated as ethylene receptors genes (*Ethylene receptor_a*, PGSC0003DMG400016284, *Ethylene receptor_b*, PGSC0003DMG400023402, *Ethylene receptor_c*, PGSC0003DMG400023402, and *Ethylene receptor homolog_b*, PGSC0003DMG400031819), whilst 1-MCP inhibited their expression (MC and ME) (Fig. 7A).

In CE tubers, ethylene strongly increased the expression of two *ACO* genes, *ACO_c* and *ACO homolog_b* (PGSC0003DMG400002321 and PGSC0PGSC0003DMG400017246, respectively), following a pattern similar to that observed during the transient respiration increase: *ACO_c* and *ACO homolog_b* expression peaked at day 3 (55- and 23-times higher than day 0, respectively), before starting to decline (Fig. 7B). No variations were identified in *ACO* genes expression among the other treatments (Fig. 7C) where there was no peak in respiration (Fig. 3B and C).

### 3.4 Ethylene responses in cortex tissue: upregulation of ABA

**Fig. 8:**
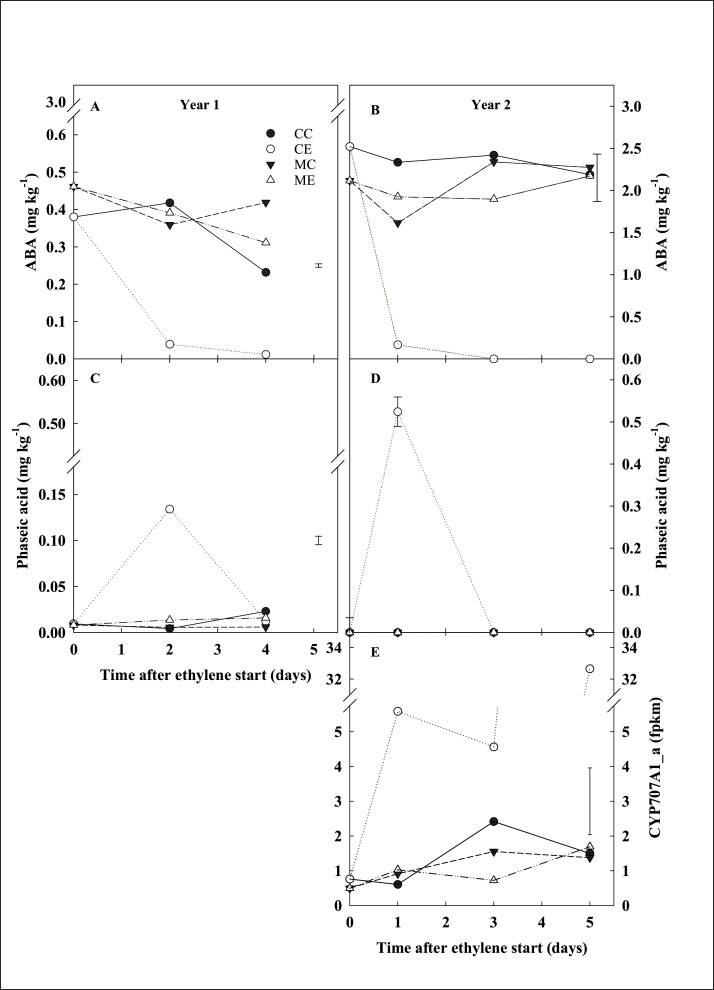
Abscisic acid (ABA. DW, mg kg^−1^) accumulation and catabolism. Cortex was sampled from cv. VR808 tubers stored at 8.5 °C under four different treatments: CC (control + control); CE (control + continuous ethylene, 10 μL L^-1^); MC (1-MCP, 1 μL L^-1^ 24 h, + control); ME (1-MCP + continuous ethylene, 10 μL L^-1^). ABA (A and B), and phaseic acid (C and D) content were determined by LC–MS/MS. Gene expression of *ABA 8ˊ-hydroxylase* gene *CYP707A1_a* is reported from RNAseq data (E), where fpkm is fragments per kilobase of transcript per million mapped reads. Least significant difference (LSD_0.05_) bars are shown (A, B, C, and E). Error bars representing the standard error were used when the interaction treatment*time was not significant (D). Axis breaks are included where required.

Variation in ABA metabolism was studied through metabolomic (LC/MS-MS) and transcriptomic approaches. Cortex samples collected during the first week of ethylene treatment across years 1 and 2 were selected (Fig. 4A). Ethylene supplementation induced a remarkably rapid decline in ABA concentrations in CE tubers within the first two days of treatment (Fig. 8A, B): CE cortex showed a 9-fold decrease in ABA by day 2 in year 1 (Fig. 8A), and a 15-fold decline by day 1 in year 2 (Fig. 8B). The rapid decrease in ABA was associated with a transient accumulation of phaseic acid in both year 1 (14-fold increase) and year 2 (detected in CE samples only) (Fig. 8C, D), while dihydrophaseic acid was not detected. Neither ME tubers, nor the other treatments (CC, MC) showed significant variations in ABA catabolism during the first week of storage. The transcriptomic analysis of ABA metabolism genes highlighted that the expression of *CYP707A1_a* (PGSC0003DMG400001960), encoding ABA 8ˊhydroxylase, was strongly upregulated in CE tubers from the beginning of storage (7-times higher at day 1 than day 0), and maintained the upregulation throughout the first week (43-fold higher at day 5 than day 0) whilst its expression was unaffected by other treatments (Fig. 8E). During the same period, transcripts from four members of the *NCED* (*9-cis-epoxycarotenoid dioxygenase*) gene family, responsible for the key rate-limiting step in ABA biosynthesis, did not show any significant difference in response to any treatment.

### 3.5 Ethylene responses in cortex tissue: upregulation of starch catabolism and reducing sugars accumulation

**Fig. 9:**
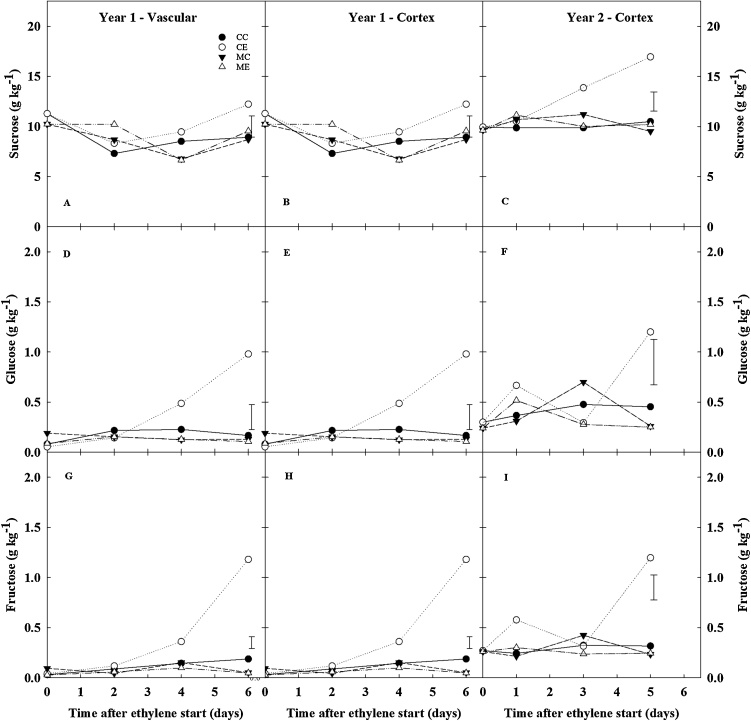
Sugar content of tuberous parenchyma. Cortex and vascular tissues were sampled from cv. VR808 tubers stored at 8.5 °C under four different treatments: CC (control + control); CE (control + continuous ethylene, 10 μL L^−1^); MC (1-MCP, 1 μL L^−1^ 24 h, + control); ME (1-MCP + continuous ethylene, 10 μL L^−1^). Sucrose (A, B, C). Glucose (D, E, F). Fructose (G, H, I). Data are on a dry weight basis. Least significant difference (LSD_0.05_) bars are shown. The expression profiles of starch and sugar metabolism genes highlighted that CE was strongly differentiated from any other treatment (CC, MC, ME), and exhibited the highest impact on gene expression (Fig. 10A).

**Fig. 10:**
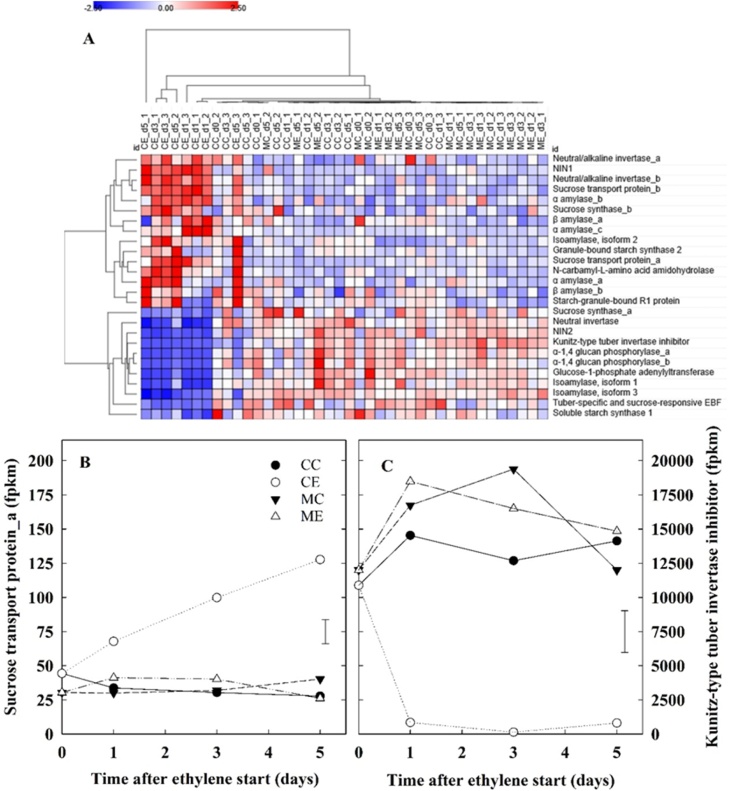
Starch and sugar metabolism. Cortex was sampled from cv. VR808 tubers stored at 8.5 °C under four different treatments: CC (control + control); CE (control + continuous ethylene, 10 μL L^−1^); MC (1-MCP, 1 μL L^−1^ 24 h, + control); ME (1-MCP + continuous ethylene, 10 μL L^−1^). Heat map of selected starch and sugar metabolism genes (A); and gene expression for *Sucrose transport protein_a* gene (B); and *Kunitz-type invertase inhibitor* gene (C). Gene expression is reported from RNAseq data, where fpkm is fragments per kilobase of transcript per million mapped reads. Least significant difference (LSD_0.05_) bars are shown. NIN*, neutral invertase.*

Sugar profiles obtained from cortex tissue during the first week of ethylene supplementation in both years 1 and 2 confirmed that CE was the only treatment able to affect sugar content. The results showed that sucrose and reducing sugars accumulated within four-five days from the start of exogenous ethylene treatment, and that sucrose accumulation occurred as a first response (Fig. 9B, C). Reducing sugars accumulation in the cortex was slightly delayed (Fig. 9E, F, H, I), as was also found in the bulk samples (Fig. 5). In year 1, sugar accumulation profiles were also studied in the isolated vascular tissue (Fig. 9A, D, G; Fig. 4B). The expression profiles of starch and sugar metabolism genes highlighted that CE was strongly differentiated from any other treatment (CC, MC, ME), and exhibited the highest impact on gene expression (Fig. 10A).

In the cortex, ethylene induced the expression of genes encoding proteins involved in starch breakdown (α-and β-amylase, and starch granule-bound R1 protein) and hydrolysis of disaccharides: *neutral invertase 1* (*NIN1;* PGSC0003DMG400009909)*, neutral/alkaline invertase_b* (PGSC0003DMG400026107). Related to the induction of invertase gene expression, a *Kunitz-type invertase inhibitor* (PGSC0003DMG400010146) exhibited a substantial reduction in expression in CE tubers (13-fold) immediately after the start of ethylene treatment and was maintained at very low expression levels throughout the first week of storage (Fig. 10C). Sucrose transporter transcripts were also induced in CE tubers (Fig. 10A, B). At the same time, ethylene negatively affected the expression of genes related to early steps of starch biosynthesis, such as those encoding glucose-1-phosphate adenylyltransferase, and α-1,4 glucan phosphorylase, and two isoamylases (Fig. 10A).

## 4 Discussion

In June 2019, the European Union decreed that CIPC would be withdrawn as a sprout suppressant for potato storage. This landmark decision was taken although the permitted Maximum Residue Levels applied have been continually reduced over the last decade. As a consequence, concomitant research on viable CIPC alternatives (*e.g.* exogenous ethylene, essential oils, controlled atmosphere) has accelerated (Alamar et al., 2017a). Among these alternatives, exogenous ethylene is well known to affect potato physiology during storage and many have reported the positive effects on sprouting delay, alongside the propensity to promote the undesirable accumulation of reducing sugars (Daniels-Lake et al., 2005, 2011; Foukaraki et al., 2014, Foukaraki et al., 2016a, 2016b; Prange et al., 1998, 2005; Suttle, 1998). 1-MCP has been tested and has showed potential in reducing ethylene-induced sugar accumulation (Daniels-Lake et al., 2008; Foukaraki et al., 2016b; Prange et al., 2005). Nevertheless, there remains a paucity of understanding over the molecular mechanisms which explain how ethylene and 1-MCP affect tuber physiology. In addition, 1-MCP has yet to be registered as a commercially approved storage technology for potato.

The current work has shown that exogenous ethylene elicited an increase in respiration activity and soluble sugars of tubers (CE), and that these pathways were triggered within a few days from the start of ethylene supplementation. In contrast, these responses were totally absent in presence of 1-MCP (ME).

### 4.1 Potatoes respond to exogenous ethylene by activating conserved pathways

Continuous ethylene supplementation induced a transient increase in respiration rate of CE tubers which lasted for *ca*. 5 days (Fig. 3). These *in situ* respiratory results were in general agreement with previous works describing a respiration peak for potato tubers within a few hours from commencement of exogenous ethylene treatment, when tubers were held at room temperature (Day et al., 1978; Huelin and Barker, 1939; Rylski et al., 1974). In the present study, the observed transient respiration peak was completely inhibited by 1-MCP (ME); this inhibition suggested that the transient respiration increase, which relates to the ethylene-induced sugar accumulation, may be analogous to that described in cold stored tubers (Duplessis et al., 1996).

Building on this, a similarly transient expression of two *ACO* genes was observed: *ACO_c* and *ACO homolog_b* (Fig. 7B). Upregulation of *ACO* gene expression, or ACO enzyme activity, following exogenous ethylene has been described for other storage organs (Cools et al., 2011; Alamar et al., 2020), but not for potato tubers. The transcriptomic profiles of ethylene receptors in this work showed that in potato cortex the expression of four receptor genes (*ethylene receptor_a*, *ethylene receptor_b*, *ethylene receptor_c* and *ethylene receptor homolog_b*) was induced by ethylene and inhibited by 1-MCP (Fig. 7A). Our results indicate that stored potato tubers, even though they are characterised by very low levels of endogenous ethylene, responded to exogenous ethylene supplementation by activating a series of conserved pathways that also exist in many plant organs.

### 4.2 Exogenous ethylene induced reducing sugar accumulation through a similar mechanism to cold-induced sweetening by down-regulating a Kunitz-type invertase inhibitor

Although it is well known that exogenous ethylene elicits the accumulation of reducing sugars (Briddon, 2006; Colgan et al.,2012; Daniels-Lake et al., 2005; Foukaraki et al., 2014, Foukaraki et al., 2016a, 2016b; Prangeet al., 1998, 2005), the mechanisms behind this ethylene-induced sweetening have not been characterised. The accumulation of reducing sugars is undesirable for processing potatoes because the Maillard reaction can lead to dark fry colour and possible acrylamide formation under high cooking temperatures. The results of the current study showed that ethylene rapidly induced a transient accumulation of reducing sugars within four to five storage days (Fig. 5, Fig. 9). Foukaraki et al. (2014, 2016b) reported that ethylene-induced sweetening was longstanding and could last several months, while the data herein highlighted that reducing sugars returned to original levels after three months of storage. Reducing sugar content is a cultivar-dependent trait affected by many factors, including storage conditions (Neilson et al., 2017): temperature below 7 °C induces so-called cold-induced sweetening (Bhaskar et al., 2010; Foukaraki et al., 2014, Foukaraki et al., 2016a, 2016b; Liu et al., 2013), while exogenous ethylene supplementation is responsible for the ethylene-induced sweetening (Daniels-Lake et al., 2005; Prange et al., 1998, 2005; Pritchard and Adam, 1994). It is clear that the invertase gene family plays a crucial role in the cold-induced accumulation of reducing sugars (Bhaskar et al., 2010; Liu et al., 2013; Mckenzie et al., 2013). The transcriptional profiles presented in the current work showed that, as soon as ethylene treatment began, starch biosynthesis and breakdown were inhibited and induced, respectively. At the same time, two invertase genes (*NIN1*, and *neutral/alkaline invertase_b*) were upregulated in CE tubers, whilst ethylene strongly inhibited a *Kunitz-type invertase inhibitor* (Fig. 10). The expression of this *Kunitz-type invertase inhibitor* has been related with cold-induced sweetening, and its overexpression decreased susceptibility to cold-induced sweetening (Liu et al., 2013). The data from the current study highlighted that the downregulation of this *Kunitz-type invertase inhibitor* was greater (13-fold lower by day 1 of storage in ethylene) than the upregulation of *NIN1* and *Neutral/alkaline invertase_b* (2.1- and 5.6-fold, respectively), suggesting a predominant role of this invertase inhibitor in the accumulation of reducing sugars. This result appears to be in agreement with Fischer et al. (2013), who reported that the Kunitz-type invertase inhibitor protein showed the highest (negative) correlation with tuber sugar content. Taken together, these findings seemed to suggest that cold-induced and ethylene-induced sweetening may be triggered by the same factors: the shift in the dynamic equilibrium between starch biosynthesis/breakdown, and the concomitant robust downregulation of the *Kunitz-type invertase inhibitor* expression. Further analyses of the expression of this *Kunitz-type invertase inhibitor* during storage, or the behaviour of over-expression lines (Liu et al., 2013), would be helpful in elucidating whether the downregulation described in the current work also plays a role in the duration of ethylene-induced sweetening. Moreover, the present work also highlights the possibility of searching for favourable natural alleles of this gene that are less responsive to external factors, such as temperature and exogenous ethylene, so as to prevent sugar accumulation.

### 4.3 Exogenous ethylene rapidly activates ABA catabolism in cortex tissue

The role of ABA in mediating potato dormancy has been well characterised in meristematic tissue and it is known that ABA accumulation reaches a maximum during tuberisation and dormancy onset, while ABA decline is a key factor in determining dormancy break (Aksenova et al., 2013; Alamar et al., 2017a; Sonnewald, 2001; Sonnewald and Sonnewald, 2014; Suttle, 2004). Despite this, the role of parenchymatic ABA in mediating dormancy has not been studied. It has been reported that tubers treated with continuous ethylene (10 μL L^−1^) exhibited increased ABA concentration in parenchymatic tissue after six weeks of storage, together with decreased amounts of dihydrophaseic acid, an ABA catabolite (Foukaraki et al., 2016a). The results of the current work showed that within 24 h from the beginning of continuous ethylene treatment, CE tubers showed a rapid and sharp decline in ABA content (15-times lower than day 0) together with a concomitant transient increase in phaseic acid (Fig. 8). In addition, exogenous ethylene upregulated the expression of *CYP707A1_a*, encoding catabolic enzyme ABA 8ˊ-hydroxylase, from the beginning of storage, while no variations were found in the expression of ABA biosynthesis genes. The results describe a clear temporal progression from an early but sustained induction of *CYP707A1_a*, followed by a temporary rise in phaseic acid, and an associated gradual decline and exhaustion of ABA. This behaviour is consistent with a rise in catabolism transcriptionally regulated by *CYP707A1_a*, combined with a low and static rate of synthesis. Destefano-Beltrán et al. (2006), investigating the expression pattern of ABA biosynthesis and catabolism genes in different tissues of potato tuber, described a constant downregulation of *CYP707A1* and *CYP707A2* expression in cortex tissue during storage. The results of the current work clearly showed that exogenous ethylene strongly induced the expression of *CYP707A1_a* in cortex (7- and 43-fold greater at days 1 and 5, respectively, compared to day 0). This observation is similar to the ethylene-induced ABA catabolism (*via CYP707A5* upregulation) reported in deep water rice during the submergence response (Saika et al., 2007; Yang and Choi, 2006).

Rylski and colleagues (1974) described a dual role of ethylene in sprouting regulation, where after a short ethylene treatment, sprouting was hastened, while following continuous ethylene sprouting was delayed; similarly, cessation of exogenous ethylene supplementation induced increased sprouting (Foukaraki et al., 2014). The ABA catabolism results presented herein might partially explain why short or interrupted ethylene treatments accelerate sprouting in potato tubers: if ABA is acting as a sprout growth inhibitor in potato sprouts, rapid depletion of ABA in response to ethylene may accelerate sprouting in the short-term, whereas longer-term effects of ethylene through different pathways may induce ecodormancy. However, it is important to recognise that the directionality of the effects of ABA on growth is dose-dependent in many systems (Humplík et al., 2017).

### 4.4 1-MCP enhances the effect of ethylene on extending dormancy and reducing sprout elongation

Exogenous ethylene inhibits sprout elongation, while ecodormancy is extended by the combination of ethylene and 1-MCP. Previous works reported that ethylene was able to extend ecodormancy in potato (Daniels-Lake et al., 2005; Foukaraki et al., 2016a, 2016b; Prange et al., 1998; Rylski et al., 1974). However, using improved phenotyping, this study has highlighted that ethylene had less effect on ecodormancy when applied alone (CE) (Fig. 5, arrows), but strongly delayed sprout elongation (Fig. 2). An analogous behaviour was recently described in transgenic lines of potato overexpressing the gene *StCEN*, a homologue of *TERMINAL FLOWER 1/CENTRORADIALIS* associated with a quantitative trait loci (QTL) for potato sprout vigour (Morris et al., 2019). Despite the phenotypical similarity, the current work did not find a link between exogenous ethylene and *StCEN* expression, which did not show any significant variations, neither related to ethylene, nor to other treatments.

Cools and colleagues (2011) reported that greater inhibition of sprout growth in onion was achieved by combining ethylene and 1-MCP. Similarly, in this study, the weekly sprout assessments highlighted that 1-MCP combined with ethylene (ME) was successful in extending ecodormancy of tubers by one-to-three weeks compared to ethylene alone (CE) (Fig. 5, arrows).

## 5 Conclusions

The current study showed for the first time that potato tubers adapt to continuous exogenous ethylene (10 μL L^-1^) supplementation by activating a series of conserved mechanisms already reported during low temperature storage adaptation. In addition, ethylene has a strong enhancer effect on parenchymatic ABA catabolism and this may explain the dual effect that ethylene can have on sprout suppression. Moreover, the results highlighted that ethylene- and cold-induced sweetening are both (inversely) related to expression of one *Kunitz-type invertase inhibitor* gene, opening the possibility for improved breeding strategies.

## Author statement

R.T. designed and performed most of the experimental work, and wrote the article with contributions from all authors. L.A.T., G.C., M.C.A., K.C., S.M. and A.J.T conceived the research plans and supervised the work; A.W. and R.T. carried out transcriptomic data analysis; L.A.T. agrees to serve as the author responsible for contact.

## Declaration of Competing Interest

Authors Waters A. is employed by PepsiCo, Inc. Authors Chope G.A. and McWilliam S. are employed by PepsiCo International Limited. The views expressed in this manuscript are those of the authors and do not necessarily reflect the views or policies of PepsiCo, Inc. or any of its affiliates.

## References

[bib0005] Aksenova N.P., Sergeeva L.I., Konstantinova T.N., Golyanovskaya S.A., Kolachevskaya O.O., Romanov G.A. (2013). Regulation of potato tuber dormancy and sprouting. Russ. J. Plant Physiol..

[bib0010] Alamar M.C., Tosetti R., Landahl S., Bermejo A., Terry L.A. (2017). Assuring potato tuber quality during storage: a future perspective. Front. Plant Sci..

[bib0015] Alamar M.C., Collings E., Cools K., Terry L.A. (2017). Impact of controlled atmosphere scheduling on strawberry and imported avocado fruit. Postharvest Biol. Technol..

[bib0020] Alamar M.C., Anastasiadi M., Lopez-Cobollo R., Bennett M.H., Thompson A.J., Turnbull C.G.N., Mohareb F., Terry L.A. (2020). Transcriptome and phytohormone changes associated with ethylene-induced onion bulb dormancy. Postharvest Biol. Technol..

[bib0025] Amoah R.S., Landahl S., Terry L.A. (2016). The timing of exogenous ethylene supplementation differentially affects stored sweetpotato roots. Postharvest Biol. Technol..

[bib0030] Amoah R.S., Landahl S., Terry L.A. (2017). Design and construction of a flexible laboratory-scale mixing apparatus for continuous ethylene supplementation of fresh produce. Biosyst. Eng..

[bib0035] Anders S., Pyl P.T., Huber W. (2015). HTSeq—a Python framework to work with high-throughput sequencing data. Bioinformatics.

[bib0040] Andrews S. (2010). FastQC: A Quality Control Tool for High Throughput Sequence Data. http://www.bioinformatics.babraham.ac.uk/projects/fastqc.

[bib0045] Barnett D.W., Garrison E.K., Quinlan A.R., Strömberg M.P., Marth G.T. (2011). BamTools: a C++ API and toolkit for analyzing and managing BAM files. Bioinformatics.

[bib0050] Bhaskar P.B., Wu L., Busse J.S., Whitty B.R., Hamernik A.J., Jansky S.H., Buell C.R., Bethke P.C., Jiang J. (2010). Suppression of the vacuolar invertase gene prevents cold-induced sweetening in potato. Plant Physiol..

[bib0055] Bolger A.M., Lohse M., Usadel B. (2014). Trimmomatic: a flexible trimmer for illumina sequence data. Bioinformatics.

[bib0060] Briddon A. (2006). The use of ethylene for sprout control. BPC Research Review, Ref..

[bib0065] Colgan R., Harper G., Taylor M., Bryan G., Rees D. (2014). Reducing Energy Usage and Wastage by Improving Ethylene Control of Potato Sprouting.

[bib0070] Collings E.R., Gavidia M.C.A., Cools K., Redfern S., Terry L.A. (2018). Effect of UV-C on the physiology and biochemical profile of fresh *Piper nigrum* berries. Postharvest Biol. Technol..

[bib0075] Cools K., Chope G.A., Hammond J.P., Thompson A.J., Terry L.A. (2011). Ethylene and 1-methylcyclopropene differentially regulate gene expression during onion sprout suppression. Plant Physiol..

[bib0080] Daniels-Lake B.J., Prange R.K., Nowak J., Asiedu S.K., Walsh J.R. (2005). Sprout development and processing quality changes in potato tubers stored under ethylene: 1. Effects of ethylene concentration. Am. J. Potato Res..

[bib0085] Daniels-Lake B.J., Prange R.K., Bishop S.D., Hiltz K. (2008). 1-methylcyclopropene counteracts fry color darkening attributable to carbon dioxide and ethylene interaction. Hort. Sci..

[bib0090] Daniels-Lake B.J., Pruski K., Prange R.K. (2011). Using ethylene gas and chlorpropham potato sprout inhibitors together. Potato Res..

[bib0095] Day D.A., Arron G.P., Christoffersen R.E., Laties G.G. (1978). Effect of ethylene and carbon dioxide on potato metabolism: stimulation of tuber and mitochondrial respiration, and inducement of the alternative path. Plant Physiol..

[bib0100] Destefano-Beltrán L., Knauber D., Huckle L., Suttle J.C. (2006). Effects of postharvest storage and dormancy status on ABA content, metabolism, and expression of genes involved in ABA biosynthesis and metabolism in potato tuber tissues. Plant Mol. Bio..

[bib0105] Duplessis P.M., Marangoni A.G., Yada R.Y. (1996). A mechanism for low temperature induced sugar accumulation in stored potato tubers: the potential role of the alternative pathway and invertase. Am. Potato J..

[bib0110] Fischer M., Schreiber L., Colby T., Kuckenberg M., Tacke E., Hofferbert H.R., Schmidt J., Gebhardt C. (2013). Novel candidate genes influencing natural variation in potato tuber cold sweetening identified by comparative proteomics and association mapping. BMC Plant Biol..

[bib0115] Foukaraki S.G., Cools K., Chope G.A., Terry L.A. (2014). Effect of the transition between ethylene and air storage on post-harvest quality in six UK-grown potato cultivars. The J. Hortic. Sci. Biotech..

[bib0120] Foukaraki S.G., Cools K., Chope G.A., Terry L.A. (2016). Impact of ethylene and 1-MCP on sprouting and sugar accumulation in stored potatoes. Postharvest Biol. Technol..

[bib0125] Foukaraki S.G., Cools K., Terry L.A. (2016). Differential effect of ethylene supplementation and inhibition on abscisic acid metabolism of potato (*Solanum tuberosum* L.) tubers during storage. Postharvest Biol. Technol..

[bib0130] Hardigan M.A., Crisovan E., Hamilton J.P., Kim J., Laimbeer P., Leisner C.P., Manrique-Carpintero N.C., Newton L., Pham G.M., Vaillancourt B., Yang X. (2016). Genome reduction uncovers a large dispensable genome and adaptive role for copy number variation in asexually propagated *Solanum tuberosum*. Plant Cell.

[bib0135] Hinchcliffe A., Barker I., Garthwaite D.G., Parrish G. (2018). Pesticide Usage Survey Report 275 - Potato Stores in the United Kingdom 2016.

[bib0140] Huelin F.E., Barker J. (1939). The effect of ethylene on the respiration and carbohydrate metabolism of potatoes. New Phytol..

[bib0145] Humplík J.F., Bergougnoux V., Van Volkenburgh E. (2017). To stimulate or inhibit? That is the question for the function of abscisic acid. Trends Plant Sci..

[bib0150] Kim D., Pertea G., Trapnell C., Pimentel H., Kelley R., Salzberg S.L. (2013). TopHat2: accurate alignment of transcriptomes in the presence of insertions, deletions and gene fusions. Genome Biol..

[bib0155] Liu X., Cheng S., Liu J., Ou Y., Song B., Zhang C., Lin Y., Li X.Q., Xie C. (2013). The potato protease inhibitor gene, St-Inh, plays roles in the cold-induced sweetening of potato tubers by modulating invertase activity. Postharvest Biol. Technol..

[bib0160] Love M.I., Huber W., Anders S. (2014). Moderated estimation of fold change and dispersion for RNA-seq data with DESeq2. Genome Biol..

[bib0165] Mckenzie M.J., Chen R.K., Harris J.C., Ashworth M.J., Brummell D.A. (2013). Post‐translational regulation of acid invertase activity by vacuolar invertase inhibitor affects resistance to cold‐induced sweetening of potato tubers. Plant Cell Environ..

[bib0170] Morris W.L., Alamar M.C., Lopez-Cobollo R.M., Castillo Cañete J., Bennett M., Van der Kaay J., Stevens J., Kumar Sharma S., McLean K., Thompson A.J., Terry L.A., Turnbull C.G.N., Bryan G.J., Taylor M.A. (2019). A member of the TERMINAL FLOWER 1/CENTRORADIALIS gene family controls sprout growth in potato tubers. J. Exp. Bot..

[bib0175] Müller M., Munné-Bosch S. (2011). Rapid and sensitive hormonal profiling of complex plant samples by liquid chromatography coupled to electrospray ionization tandem mass spectrometry. Plant Methods.

[bib0180] Neilson J., Lagüe M., Thomson S., Aurousseau F., Murphy A.M., Bizimungu B., Deveaux V., Bègue Y., Jacobs J.M.E., Tai H.H. (2017). Gene expression profiles predictive of cold-induced sweetening in potato. Funct. Integr. Genomics.

[bib0185] Ohanenye I.C., Alamar M.C., Thompson A.J., Terry L.A. (2019). Fructans redistribution prior to sprouting in stored onion bulbs is a potential marker for dormancy break. Postharvest Biol. Technol..

[bib0190] Prange R.K., Kalt W., Daniels-Lake B.J., Liew C.L., Page R.T., Walsh J.R., Dean P., Coffin R. (1998). Using ethylene as a sprout control agent in stored ‘Russett Burbank’ potatoes. J. Am. Soc. Hortic. Sci..

[bib0195] Prange R.K., Daniels-Lake B.J., Jeong J., Binns M. (2005). Effects of ethylene and 1-methylcyclopropene on potato tuber sprout control and fry color. Am. J. Potato Res..

[bib0200] Pritchard M.K., Adam L.R. (1994). Relationships between fry color and sugar concentration in stored Russet Burbank and Shepody potatoes. Am. Potato J..

[bib0205] Rylski I., Rappaport L., Pratt H.K. (1974). Dual effects of ethylene on potato dormancy and sprout growth. Plant Physiol..

[bib0210] Saika H., Okamoto M., Miyoshi K., Kushiro T., Shinoda S., Jikumaru Y., Fujimoto M., Arikawa T., Takahashi H., Ando M., Arimura S.I. (2007). Ethylene promotes submergence-induced expression of OsABA8ox1, a gene that encodes ABA 8′-hydroxylase in rice. Plant Cell Physiol..

[bib0215] Sonnewald U. (2001). Control of potato tuber sprouting. Trends Plant Sci..

[bib0220] Sonnewald S., Sonnewald U. (2014). Regulation of potato tuber sprouting. Planta.

[bib0225] Suttle J.C. (1998). Involvement of ethylene in potato microtuber dormancy. Plant Physiol..

[bib0230] Suttle J.C. (2004). Physiological regulation of potato tuber dormancy. Am. J. Potato Res..

[bib0235] Untergasser A. (2008). RNA Miniprep Using CTAB. Untergasser’s Lab Summer. http://www.untergasser.com/lab/protocols/miniprep_rna_ctab_v1_0.htm.

[bib0240] Yang S.H., Choi D. (2006). Characterization of genes encoding ABA 8′-hydroxylase in ethylene-induced stem growth of deepwater rice (*Oryza sativa* L.). Biochem. Biophys. Res. Commun..

