# Supplementary material for: New insights into the effects of ethylene on ABA catabolism, sweetening and dormancy in stored potato tubers
Source: Postharvest Biol Technol. 2021 Mar;173:111420. doi: 10.1016/j.postharvbio.2020.111420 (PMC7814342; doi:10.1016/j.postharvbio.2020.111420)
Supplement: Supplementary file 1 [file mmc1.pdf]

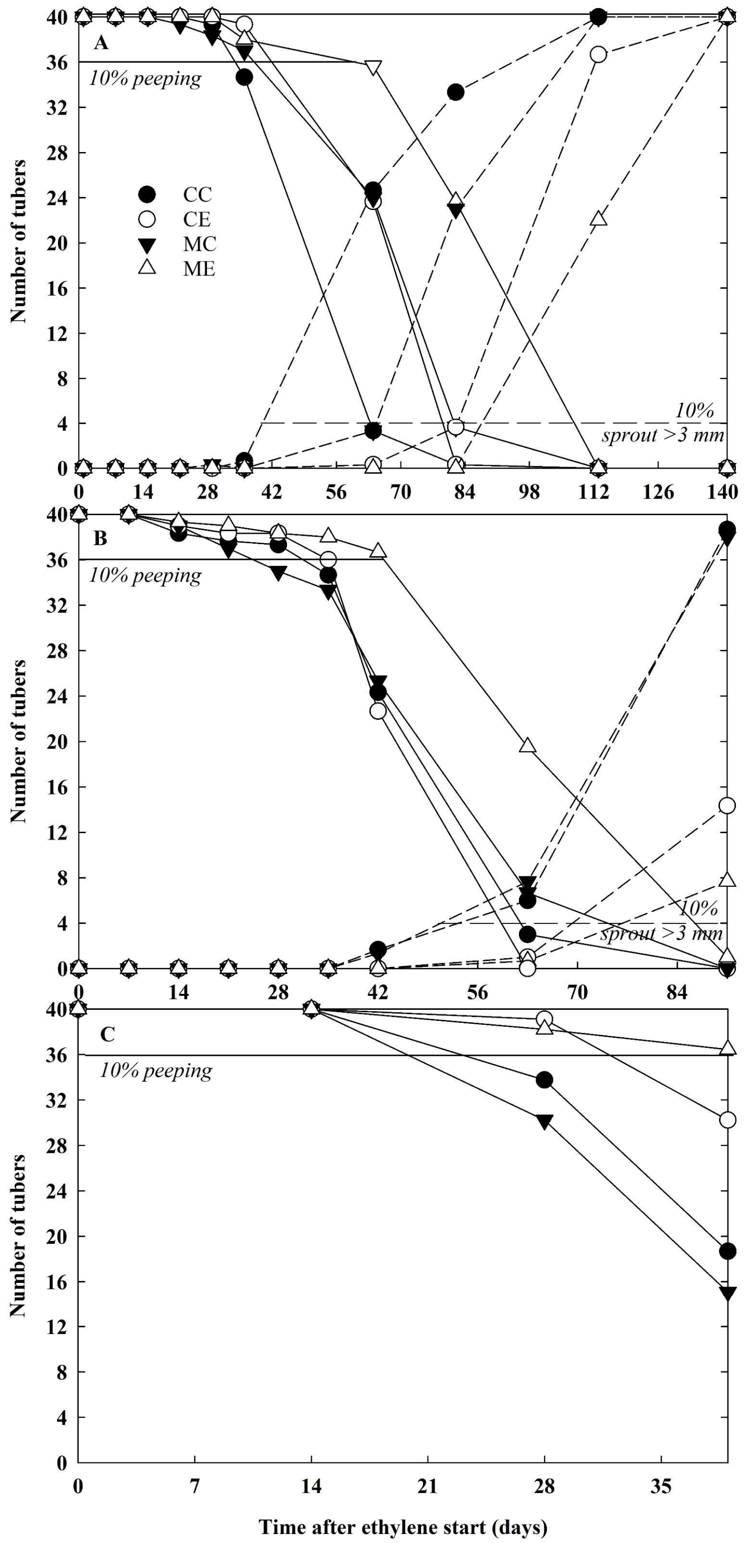

Supplemental Figure 1. Sprout assessment. Tubers from cv. VR808 tubers were stored at 8.5°C under four different treatments: CC (control + control); CE (control + continuous ethylene, 10 μL L^{-1}); MC (1-MCP, 1 μL L^{-1} 24h, + control); ME (1-MCP + ethylene, 10 μL L^{-1}). Continuous black line on the left indicates dormancy break (10% peeping). Dotted line on the right indicate elongation of sprout (sprout > 3 mm).

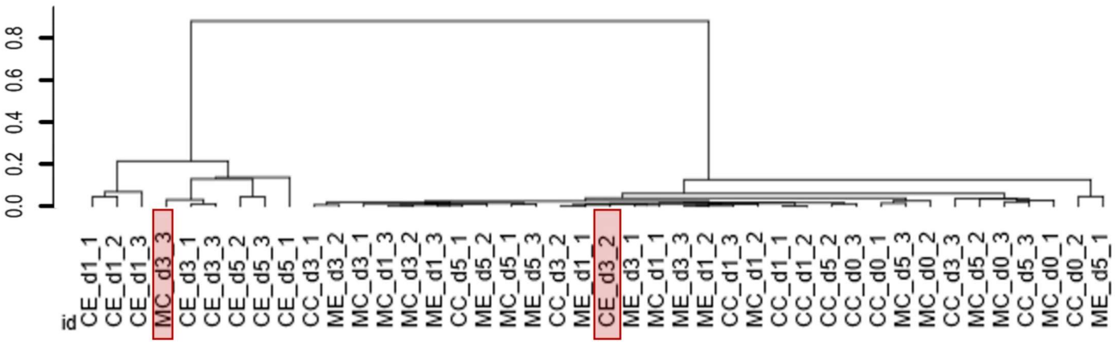

Supplemental Figure 2. Hierarchical clustering of the complete normalised transcriptomic dataset. Cortex was sampled from cv. VR808 tubers stored at 8.5°C under four different treatments: CC (control + control); CE (control + continuous ethylene, 10 μL L^{-1}); MC (1-MCP, 1 μL L^{-1} 24h, + control); ME (1-MCP + continuous ethylene, 10 μL L^{-1}). Samples were collected at day 0 (d0), 1 (d1), 3 (d3), 5 (d5), from ethylene start. Underscore numbers indicate the biological replicate number. Highlighted samples were excluded from further analysis.

Supplemental table 1. Analysed genes belonging to ethylene, abscisic acid, starch-sugar metabolism pathways.

**Ethylene**	
PGSC0003DMG400012186	ACS_a
PGSC0003DMG400021651	ACS4_a
PGSC0003DMG400033885	ACS4_b
PGSC0003DMG400003918	ACS_b
PGSC0003DMG400031027	ACS_c
PGSC0003DMG400020070	ACO_a
PGSC0003DMG400021476	ACO_b
PGSC0003DMG400017190	ACO homolog_a
PGSC0003DMG400017246	ACO homolog_b
PGSC0003DMG400002321	ACO_c
PGSC0003DMG400016284	Ethylene receptor_a
PGSC0003DMG400017186	Ethylene receptor_b
PGSC0003DMG400023402	Ethylene receptor_c
PGSC0003DMG400007843	ETR1
PGSC0003DMG400027651	ETR2
PGSC0003DMG400028698	Ethylene receptor homolog_a
PGSC0003DMG400031819	Ethylene receptor homolog_b
**Starch-sugar**	
PGSC0003DMG400009257	Neutral/alkaline invertase_a
PGSC0003DMG400010146	Kunitz-type tuber invertase inhibitor
PGSC0003DMG400026107	Neutral/alkaline invertase_b
PGSC0003DMG400026530	Neutral invertase
PGSC0003DMG400009909	NIN1
PGSC0003DMG400013140	NIN2
PGSC0003DMG400016730	Sucrose synthase_a
PGSC0003DMG400031046	Sucrose synthase_b
PGSC0003DMG400009213	Sucrose transport protein_a
PGSC0003DMG400025610	Sucrose transport protein_b
PGSC0003DMG400000169	β amylase_a
PGSC0003DMG400000954	Isoamylase, isoform 2
PGSC0003DMG400001328	Granule-bound starch synthase 2
PGSC0003DMG400002479	α-1,4 glucan phosphorylase_a
PGSC0003DMG400003495	α-1,4 glucan phosphorylase_b
PGSC0003DMG400007974	α amylase_a
PGSC0003DMG400015115	N-carbamyl-L-amino acid amidohydrolase
PGSC0003DMG400020603	α amylase_b
PGSC0003DMG400020699	Isoamylase, isoform 1
PGSC0003DMG400024145	β amylase_b
PGSC0003DMG400031084	Glucose-1-phosphate adenylyltransferase

PGSC0003DMG401017626	α amylase_c
PGSC0003DMG402007274	Isoamylase, isoform 3
PGSC0003DMG402018552	Soluble starch synthase 1
PGSC0003DMG400007677	Starch-granule-bound R1 protein
PGSC0003DMG400003316	Tuber-specific and sucrose-responsive EBF
**ABA**	
PGSC0003DMG400001960	CYP707A1_a
